# Epidural Pulsation Accelerates the Drainage of Brain Interstitial Fluid

**DOI:** 10.14336/AD.2022.0609

**Published:** 2023-02-01

**Authors:** Xianjie Cai, Qingyuan He, Wei Wang, Chunlin Li, Hui Wang, Feng Yin, Tong Li, Dongsheng Kong, Yanxing Jia, Hongfeng Li, Junhao Yan, Xunbin Wei, Qiushi Ren, Yajuan Gao, Shuangfeng Yang, Huaiyu Tong, Yun Peng, Hongbin Han

**Affiliations:** ^1^Institute of Medical Technology, Peking University Health Science Center, Beijing, China.; ^2^Department of Radiology, Peking University Third Hospital, Beijing, China.; ^3^Beijing Key Laboratory of Magnetic Resonance Imaging Equipment and Technique, Beijing, China.; ^4^Department of Radiology, Beijing Rehabilitation Hospital, Capital Medical University, Beijing, China.; ^5^School of Biomedical Engineering, Capital Medical University, Beijing, China.; ^6^Department of Neurosurgery, Aerospace Center Hospital, Peking University Aerospace Clinical College, Beijing, China.; ^7^Department of Neurosurgery, First Medical Center, General Hospital of Chinese PLA, Beijing, China.; ^8^State Key Laboratory of Natural and Biomimetic Drugs, School of Pharmaceutical Sciences, Peking University, Beijing, China.; ^9^Department of Anatomy and Histology, School of Basic Medical Sciences, Peking University, Beijing, China.; ^10^Department of Biomedical Engineering, College of Future Technology, Peking University, Beijing, China.; ^11^Department of Radiology, Beijing Children's Hospital, Capital Medical University, National Center for Children's Health, Beijing, China.; ^12^Institute of Biomedical Engineering, Peking University Shenzhen Graduate School, Shenzhen, China.

**Keywords:** extracellular space, interstitial fluid, cerebrospinal fluid, MRI, brain

## Abstract

Unhindered transportation of substances in the brain extracellular space (ECS) is essential for maintaining brain function. Regulation of transportation is a novel strategy for treating ECS blockage-related brain diseases, but few techniques have been developed to date. In this study, we established a novel approach for accelerating the drainage of brain interstitial fluid (ISF) in the ECS using minimally invasive surgery, in which a branch of the external carotid artery is separated and implanted epidurally (i.e., epidural arterial implantation [EAI]) to promote a pulsation effect on cerebrospinal fluid (CSF) in the frontoparietal region. Tracer-based magnetic resonance imaging was used to evaluate the changes in ISF drainage in rats 7 and 15 days post-EAI. The drainage of the traced ISF from the caudate nucleus to ipsilateral cortex was significantly accelerated by EAI. Significant increases in the volume fraction of the ECS and molecular diffusion rate were demonstrated using the D_ECS_-mapping technique, which may account for the mechanisms underlying the changes in brain ISF. This study provides a novel perspective for encephalopathy treatment via the brain ECS.

Due to the absence of a lymphatic system in the brain [1], substance transport and elimination of metabolic waste rely predominantly on interstitial fluid (ISF) drainage. Perturbations of ISF drainage may cause abnormal deposition of substances in the brain extracellular space (ECS), resulting in brain dysfunction such as Alzheimer's disease (AD) [2-4], stroke [5, 6], and epilepsy [7]. Accordingly, improving brain ISF drainage has been proposed as a promising therapeutic strategy for brain diseases [1, 8-10]. Our previous study verified that the deposition of amyloid β (Aβ) protein in the AD brain leads to ECS blockage and a reduction in ISF drainage, and red light at 630 nm disrupts Aβ deposition in the ECS and recovers ISF flow accompanied by reversed memory in an AD animal model [11]. Hence, the artificial and active regulation of brain ISF drainage to promote the clearance of toxins from the brain ECS is a promising strategy for treating brain diseases.

Substance transport in the brain ECS is influenced by multiple factors. Maturation of the myelin sheath determines the direction and rate of ISF drainage from the caudate nucleus (Cn) to the ipsilateral superficial cortex [12]. Brain ISF drains bidirectionally from the Cn toward the cortex and thalamus in juvenile rats. In contrast, in the adult brain, it drains only toward the ipsilateral cortex along the mature myelin sheath [12, 13]. The exchange of ISF-cerebrospinal fluid (CSF) occurs in the superficial cortex with the assistance of arterial pulsation and then enters the subarachnoid space (SAS) [14], which then drains to the deep cervical lymph nodes via the epidural lymph vessels and flows into the somatic circulation [15]. The release of excitatory transmitters after neuronal excitation in the thalamus secondary to nociceptive stimulation leads to restricted diffusion in the ECS [16], which can result in downregulation of brain ISF drainage in the thalamus.

Based on recent findings regarding the drainage pathway and influencing factors, we hypothesized that ISF drainage may be accelerated by a change in local CSF flow. To test this theory, we achieved local arterial pulsation effects by implanting a separate arterial branch of the external carotid artery in the space between the dura and skull, and CSF acceleration enhanced the exchange of CSF-ISF, thereby altering ISF drainage in the brain parenchyma. Tracer-based magnetic resonance imaging (MRI) [17] was employed to measure alterations in ISF drainage from the Cn in rats on days 7 and 15 after epidural arterial implantation (EAI) surgery. The D_ECS_ mapping technique [18] was applied to quantitatively analyze ECS structure and molecular diffusion changes in the Cn region to clarify the microstructural basis of altered ISF drainage by EAI. Further, we discuss the significance of accelerating ISF drainage for the treatment of neurological disorders.

## MATERIALS AND METHODS

### Experimental animals

Experiments were performed on adult male Sprague-Dawley rats (8 weeks old, weighing 250-300 g). The animal experimental protocol was approved by the Peking University Biomedical Ethics Committee (permit number: LA20200335). Animals were housed under a 12-hour light/dark diurnal cycle, temperature of 22±1°C, and humidity of 60±5%. Animals were provided *ad libitum* access to food and clean drinking water [13].

Rats were randomly divided into six groups according to the surgical procedure and time interval between the experimental operation and surgery: the EAI groups (EAI 7 and EAI 15 groups), control groups (Con 7 and Con 15 groups), EAI contralateral measurement group (EAI-C7 group), and EAI plus gelatin sponge pad group (EAI-G7 group) (*n* = 12 per group for Morris water maze (MWM), *n* = 6 per group for other experiments).

#### Epidural arterial implantation

**Figure 1.**
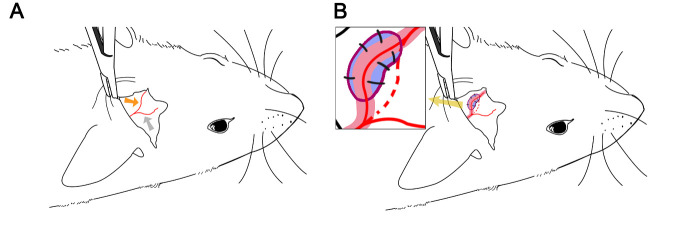
Illustration of EAI surgery. (A) The upper epidermis of the rat ear was cut, and the superficial fascia was exposed. The parietal branch (orange arrow) and frontal branch (gray arrow) of the superficial temporal artery were visible. (B) Magnification of surgery area (pointed to by yellow arrow) was shown in the top-left of the image. After the superficial temporal artery (red) and the surrounding soft tissue (pink) were successfully dissociated, the dura mater (blue) was exposed by drilling in the adjacent parietal bone area. The superficial temporal artery was affixed to the dura mater and the suture line (black line) was used to suture the soft tissue around the superficial temporal artery and the skull periosteum (purple line).

*EAI surgery:* After induction of anesthesia using intraperitoneal injection of a combination of pentobarbital sodium (Sigma, St. Louis, MO, USA); ethanol (Zhenyuminsheng Pharmaceutical Company, Beijing, China); chloral hydrate (Sigma), magnesium sulfate (Zhenyuminsheng Pharmaceutical Company); and propylene glycol (Zhenyuminsheng Pharmaceutical Company) (0.3 mL/kg) [17], each rat was placed in the prone position, and the right periotic region was shaved. The scope for shaving was positioned anterior to the area between the eyes, posterior to the neck, left of the central line, and right to approximately 1 cm below the ear. Under the microscope, the skin was cut perpendicular to the external auditory canal using microsurgery forceps and scissors to expose the temporalis. An “L”-shaped turn was made anteriorly to completely expose the temporalis and part of the skull on the top of the head. The superficial temporal artery was completely dissociated along the course of the blood vessels, fine branches were disconnected, and peripheral fascia was preserved. After dissociation, the skull at the top of the head was removed using a craniotomy drill. The skull window was extended vertically, with a length and width of approximately 2 and 1 cm, respectively. The dura was exposed along the long axis. After surgery, the skin was sutured using dislocation and relaxation sutures such that the dissociated superficial temporal artery covered the dura mater. The remaining soft tissues on both sides of the blood vessels were sutured to the residual periosteal margins on both sides, and the skin was sutured using a whole-layer suture. The skin was sterilized by wiping with iodophor, indicating completion of surgery. An illustration of the EAI surgery procedure is presented in Figure 1.

*EAI plus subvascular gelatin sponge pad procedure:* The procedure was performed in the same manner as that for the EAI surgical group. After exposing the intact dura, a piece of gelatin sponge (Xiang En Medical Technology Development Company, Beijing, China), approximately 0.5×0.5 cm in size, was carefully placed on the dura. The artery was then placed on a gelatin sponge. The skin was sterilized by wiping with iodophor, indicating the completion of surgery.

*Sham surgery:* After induction of anesthesia by intraperitoneal injection of compound anesthesia, each rat was placed in the prone position, and the right periotic region was shaved. The scope was placed anterior to the area between the eyes, posterior to the neck, left of the central line, and right to approximately 1 cm below the ear. The intact dura was exposed along the long axis. The skin was sterilized by wiping with iodophor, indicating the completion of surgery.

*Intracranial tracer injection:* Gadolinium diethyltriaminepentaacetic acid (Gd-DTPA)-enhanced MRI scanning technology, independently invented by our laboratory, was used to measure the distribution of the contrast agent in the brain ECS. Gd-DTPA (10 mmol/L, Magnevist; Bayer Schering Pharma AG, Berlin, Germany) was diluted to 10 mmol/L in 154 mmol/L NaCl solution and injected into the Cn. Rats were administered compound anesthesia. After induction of anesthesia, rats were scanned using MRI to determine the location of the injection site and then placed in a stereotaxic-single frame (Lab Standard Stereotaxic-Single; Stoelting Co., Wood Dale, IL, USA) for complete fixation. After surgical exposure of the skull, the skull was drilled according to the desired location, and Gd-DTPA was injected into the right Cn (AP: 1 mm, L: -3 mm, V: -6 mm) using a microinjection pump. For rats in the EAI-C group, Gd-DTPA was injected into the left Cn (AP: 1 mm; L: 3 mm; V: -6 mm). After correct placement of the 10-μL microsyringe (Hamilton Bonaduz AG, Bonaduz, Switzerland), the injection parameters of the syringe pump (Harvard Equipment, Holliston, MA, USA) were adjusted to deliver a total injection volume of 2 μL at an injection rate of 0.2 μL/min. After injection of the contrast agent, the needle was left in place for 10 min to prevent backflow of the contrast agent along the needle track. After 10 min, the needle was withdrawn at a uniform speed over the course of 5 min.

#### Tracer-based MRI scanning

Images were acquired using an MRI scanner (Magnetom Trio, Siemens Medical Solutions, Erlangen, Germany) equipped with a dedicated coil for small animals. Images of the rat brain were obtained using T1-weighted magnetization for rapid acquisition of gradient-echo sequences. The acquisition parameters were set as follows: echo time, 3.7 ms; repetition time, 1,500 ms; flip angle, 9°; inversion time, 900 ms; field of view, 267 mm; voxel size, 0.5 mm^3^; matrix, 512 × 512; average, 2; and phase-encoding step, 96. The acquisition time was 290 s for each rat each time point. All rats were scanned before and after tracer administration. The scan time points were pre-injection scans and 15, 30, 45, 60, 90, 120, 150, 180, and 240 min post-injection. Tracer-based MRI scans were performed on rats in the Con 7, EAI 7, EAI-C7, and EAI-G7 groups on the 7th day after surgery and on rats in the Con15 and EAI15 groups on the 15th day after surgery.

#### Brain ISF drainage and brain ECS parameter acquisition and analysis

Imaging data were processed using the Nano-Detect Analysis System software, as previously described (MRI Lab, Beijing, China) [18, 19]. All images were initially processed with grayscale calibration and histogram equalization. Subsequently, post-injection images were compared with the baseline image for image registration. Rigid transformation, mutual information measurements, high-order interpolation, and adaptive stochastic gradient descent optimization were used to achieve an accurate image registration. The images were then subtracted from the pre-scanned images. The "bright regions" obtained by creating a seed point and threshold in the region of interest were considered relevant to the tracer. A new set of post-processed MRI images was created in the horizontal, sagittal, and coronal planes. The software generated new MRI images in the horizontal, sagittal, and coronal planes, with a slice thickness of 1 mm.

The equivalent diffusion coefficient for each MRI image pixel in the vicinity of the injection site (1-2 mm from the injection site) was derived using a modified diffusion equation. The volume fraction (α) was defined as the ECS volume fraction of the entire brain tissue. Tortuosity (λ) was defined as D/D^*^, where D^*^ was the effective diffusion coefficient of a given molecule in the brain ECS, D was the diffusion coefficient of the same molecule in a free medium, and λ was the hindrance of diffusion by the local ECS structure [14]. The obtained ECS microstructure parameters included α, λ, and D^*^.

#### Physiological parameter acquisition

The heart and respiratory rates of the rats were acquired using an MR-compatible small animal monitoring and gating system (Model 1030, Small Animal Instruments, Inc., NY, USA). Measurements were made on rats in the Con 7, EAI 7, EAI-C7, and EAI-G7 groups on the 7th day post-surgery.

Animals were anesthetized as described above and were placed on the test bench. Detection needles were inserted into the two front paws and right rear paw, and a heart rate detection patch was placed on the abdomen of the rats. After commencement of measurements, data were recorded after the measurements had stabilized, and the heart and respiratory rates of the rats were recorded every 30 min. The total monitoring duration was 2 h.

Weights and local cerebral blood flow in the surgical area of rats in the Con 7, EAI 7, EAI-C7, and EAI-G7 groups (i.e., the same rats used for MRI scanning) were acquired using a balance scale (ZS-DZC, Zhongshi Dichuang Technology Development Co., Ltd., Beijing, China) and laser Doppler flowmetry (LDF, Moor VMS-LDF2, UK), respectively, pre-surgery and 7 days post-surgery.

After exposure of the intact dura during surgery, a VP4 probe of the LDF instrument was positioned approximately 1 mm from the dura. Measurements were obtained continuously for 2 min to collect pre-surgical data. After the artery covered the dura, the VP4 probe was repositioned approximately 1 mm from the dura, and measurements were obtained continuously for 2 min to collect post-surgical data.

#### Behavioral and cognitive assessments

A modified neurological severity score (mNSS) test was conducted to evaluate neurological function of rats in the Con 7 and EAI 7 groups pre-surgery and on the 7th day post-surgery. The assessments comprised motor, sensory, reflex, and balance tests, with scores ranging from 0 to 18 (normal score, 0; maximal deficit score, 18). Higher test scores indicated more severe neurological deficits [20, 21].

The MWM test was conducted to assess spatial learning and memory capability of rats in the Con 7 and EAI 7 groups as previously described [13, 22]. On day 1, rats were pretrained to find a visual platform, which was 1 cm above the surface of the water, to adapt to the test procedure. From the 2nd to 5th days, the rats were allocated 60 s and released into water from four starting points in four quadrants. The time taken by the rats to find the hidden platform submerged 1 cm below the water was recorded as the escape latency. After the platform was removed on the 6th day, the rats were released from a randomly determined starting point and allowed to swim for 60 s. The average time to find the platform (escape latency), passing times, and swim path for each rat were recorded. Test parameters were recorded automatically using a video tracking system (Zhongshi Dichuang Technology Development Co., Ltd., Beijing, China).

#### Statistical analysis

Statistical analyses were performed using SPSS version 26.0 (IBM Corp., Armonk, NY, USA). Data that followed the normal distribution were expressed as the mean ± standard deviation. Independent-samples t-tests were used for comparisons between two groups; one-way ANOVA and the Bonferroni test were used for comparisons between multiple groups. Two-way ANOVA and the Bonferroni test were used for comparisons of escape latency in MWM. Data that did not follow a normal distribution were expressed as medians and quartiles. Kruskal-Wallis test was used for comparisons between multiple groups. All statistical tests were two-sided, and a *p*-value < 0.05 was considered statistically significant.

## RESULTS

### Changes in ISF drainage on the 7th day after EAI

**Figure 2.**
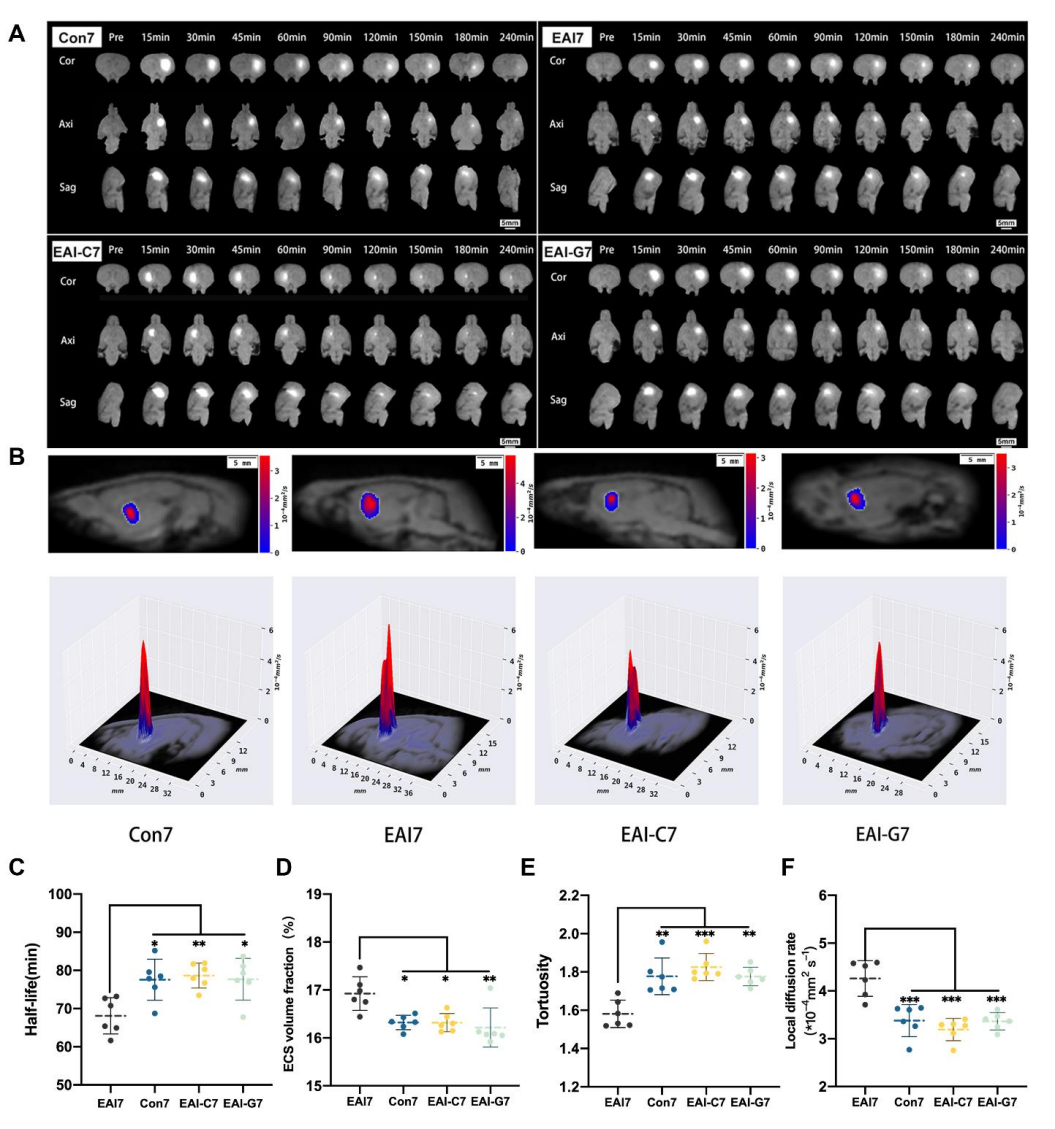
Images and comparison of ISF drainage and ECS structure parameters of rats at day 7. (A) Images of ISF drainage by tracer-based MRI scanning of the Con 7, EAI 7, EAI-C7, and EAI-G7 groups. The tracer injected into the caudate nucleus (highlighted area in the figure) spread with time to the ipsilateral cerebral cortex and eventually dissipated from the brain parenchyma (Cor: coronal image; Axi: axial image; Sag: sagittal image). Scale bar (white) = 5 mm. (B) Two-dimensional and three-dimensional mapping of local diffusion rates in ECS of Con 7, EAI 7, EAI-C7, and EAI-G7 groups. The image shows that the local diffusion rate in the EAI 7 group is higher than that in the Con 7, EAI-C7, and EAI-G7 groups. (C) Half-life of ISF drainage was lower in the EAI 7 group than that in the Con 7, EAI-C7, and EAI-G7 groups. No significant differences were observed in the comparison of the Con 7, EAI-C7, and EAI-G7 groups. D: ECS volume fraction was higher in the EAI 7 group than that in the Con 7, EAI-C7, and EAI-G7 groups. No significant differences were observed in the comparison of the Con 7, EAI-C7, and EAI-G7 groups. (E) ECS tortuosity was lower in the EAI 7 group than that in the Con 7, EAI-C7, and EAI-G7 groups. No significant differences were observed between the Con 7, EAI-C7, and EAI-G7 groups. F: Local diffusion rate in the ECS was higher in the EAI 7 group than that in the Con 7, EAI-C7, and EAI-G7 groups. No significant differences were found between the Con 7, EAI-C7, and EAI-G7 groups. Kruskal-Wallis test was used for comparisons of ECS volume fraction, ANOVA with Bonferroni test was used for comparisons of other parameters, *n* = 6 per group. *p < 0.05, **p < 0.01, ***p < 0.001 compared to the EAI 7 group. Con 7: control group (7 days); EAI 7: epidural arterial implantation group (7 days); EAI-C7: epidural arterial implantation-contralateral measurement group (7 days); EAI-G7: epidural arterial implantation-gelatin sponge group (7 days); ISF, interstitial fluid; ECS, extracellular space

Tracer-based MRI examinations revealed a tracer-labeled, high-brightness ISF from the Cn that gradually drained along the white matter to the ipsilateral cortex over time and eventually dissipated into the SAS (Fig. 2A).

Compared with the Con 7, EAI-C7, and EAI-G7 groups, the EAI 7 group exhibited faster ISF drainage from the Cn to ipsilateral cortex via the white matter (EAI 7 vs Con 7: 68.10 ± 4.75 min vs 77.57 ± 5.38 min, *p* < 0.05; EAI 7 vs EAI-C7: 68.10 ± 4.75 min vs 78.66 ± 3.20 min, *p* < 0.01; EAI 7 vs EAI-G7: 68.10 ± 4.75 min vs 77.69 ± 5.45 min, *p* < 0.05, Bonferroni test, *n* = 6). No significant difference was observed in the comparison of brain ISF drainage between the Con 7, EAI-C7, and EAI-G7 groups (77.57 ± 5.38 min vs 78.66 ± 3.20 min vs 77.69 ± 5.45 min, *p* > 0.05, Bonferroni test, *n* = 6) (Table 1, Fig. 2C).

**Table 1:** Comparison of ISF drainage and ECS structure on the 7^th^ and 15^th^ day after EAI (*n* = 6 per group).

Time	Group	T_1/2_ (min)	α (%)	λ	D* (×10^-4^ mm^2^/s)
Day 7	EAI 7	68.10 ± 4.75	16.93 ± 0.35	1.58 ± 0.70	4.26±0.37
	Con 7	77.57 ± 5.38^*^	16.32 ± 0.15^*^	1.78 ± 0.10^**^	3.38±0.34^***^
	EAI-C7	78.66 ± 3.20^**^	16.32 ± 0.19^*^	1.83 ± 0.71^***^	3.19±0.23^***^
	EAI-G7	77.69 ± 5.45^*^	16.08(16.01, 16.36)^**^	1.78 ± 0.48^**^	3.37±0.18^***^
Day 15	EAI15	67.42 ± 7.98 78.03 ± 8.35^#^	16.99 ± 0.63 16.16 ± 0.08^#^	1.56 ± 0.86 1.78 ± 0.55^###^	4.41±0.49 3.36±0.21^#^
	Con15				

Compared to the Con 7, EAI-C7, and EAI-G7 groups, the EAI 7 group exhibited accelerated ISF drainage, expanded brain ECS, decreased tortuosity, and enhanced diffusion rate in the ECS of the Cn. Compared to the Con15 group, the EAI15 group exhibited accelerated ISF drainage, expanded brain ECS, decreased tortuosity, and enhanced diffusion rate in the ECS of the Cn. No statistically significant difference was noted between the EAI 7 and EAI15 groups. Kruskal-Wallis test was used for comparisons of ECS volume fraction; ANOVA with Bonferroni test was used for comparisons of other parameters, *n* = 6 per group.

* *p* < 0.05,

** *p* < 0.01,

*** *p* < 0.001 compared to EAI 7 group;

# *p* < 0.05,

### *p* < 0.001 compared to EAI15 groupCon 7, control group (7 days); EAI 7, epidural arterial implantation group (7 days); EAI-C7, epidural arterial implantation-contralateral measurement group (7 days); EAI-G7, epidural arterial implantation-gelatin sponge group (7 days); Con15, control group (15 days); EAI15, epidural arterial implantation group (15 days); ISF, interstitial fluid; ECS, extracellular space; Cn, caudate nucleus

*Changes in brain ECS on the 7th day after EAI*

D_ECS_ mapping revealed alterations in the ECS structure and local diffusion rate within the ECS of Cn (Fig. 2B). Compared with the Con 7, EAI-C7, and EAI-G7 groups, the EAI 7 group exhibited a higher volume fraction of brain ECS [EAI 7 vs Con 7: 16.93 ± 0.35% vs 16.32 ± 0.15%, *p* < 0.05; EAI 7 vs EAI-C7: 16.93 ± 0.35% vs 16.32 ± 0.19%, *p* < 0.05; EAI 7 vs EAI-G7: 16.93 ± 0.35% vs 16.08% (16.01%, 16.36%), *p* < 0.01, Kruskal-Wallis test, *n* = 6]. No significant differences were observed in the volume fraction of ECS between the Con 7, EAI-C7, and EAI-G7 groups [16.32 ± 0.15% vs 16.32 ± 0.19% vs 16.08% (16.01%, 16.36%), *p* > 0.05, Kruskal-Wallis test, *n* = 6] (Table 1, Fig. 2D).

Compared to the Con 7, EAI-C7, and EAI-G7 groups, the EAI 7 group exhibited lower ECS tortuosity in the Cn (EAI 7 vs Con 7: 1.58 ± 0.70 vs 1.78 ± 0.10, *p* < 0.01; EAI 7 vs EAI-C7: 1.58 ± 0.70 vs 1.83 ± 0.70, *p* < 0.001; EAI 7 vs EAI-G7: 1.58 ± 0.70 vs 1.78 ± 0.48, *p* < 0.01; Bonferroni test, *n* = 6). There was no difference in the comparison of ECS tortuosity between the Con 7, EAI-C7 and EAI-G7 groups (1.78 ± 0.10 vs 1.83 ± 0.70 vs 1.78 ± 0.48, *p* > 0.05, Bonferroni test, *n* = 6) (Table 1, Fig. 2E).

Compared to the Con 7, EAI-C7, and EAI-G7 groups, the EAI 7 group exhibited a higher local diffusion rate in the Cn region (EAI 7 vs Con 7: 4.26 ± 0.37 vs 3.38 ± 0.34×10^-4^/mm^2^, *p* < 0.001; EAI 7 vs EAI-C7: 4.26 ± 0.37 vs 3.19 ± 0.23×10^-4^/mm^2^, *p* < 0.001; EAI 7 vs EAI-G7: 4.26 ± 0.37×10^-4^/mm^2^ vs 3.37 ± 0.181, *p*<0.001, Bonferroni test, *n* = 6). No significant differences were observed in the comparison of local diffusion rate within the ECS between the Con 7, EAI-C7, and EAI-G7 groups (3.38 ± 0.34 vs 3.19 ± 0.23 vs 3.37 ± 0.181×10^-4^/mm^2^, *p* > 0.05, Bonferroni test, *n* = 6) (Table 1, Fig. 2F).

### Changes to physiological and neurobehavioral function after EAI

**Figure 3.**
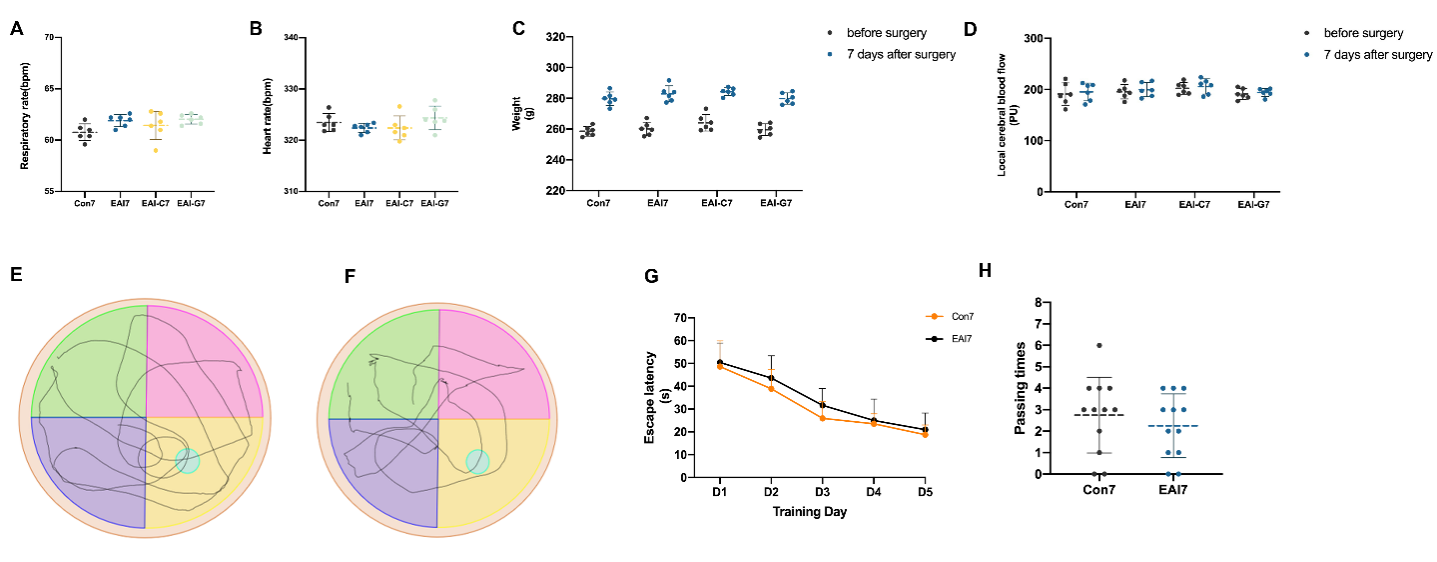
Comparison of physiological and neurobehavioral function parameters. (A-B) No significant differences were observed in respiratory and heart rates between the Con 7, EAI-C7, and EAI-G7 groups on the 7th day post-surgery. (C-D) No significant differences were observed in weights and local cerebral blood flow before and 7 days after surgery between the Con 7, EAI 7, EAI-C7, and EAI-G7 groups. (E) Tracks in the Morris water maze tests of Con 7 group. (F) Tracks in the Morris water maze tests of EAI 7 group. (G) The escape latency of the rats during a five-day period. No significant differences were observed between the Con 7 and EAI 7 groups. (H) No significant differences were observed in number of platform crossings between the Con 7 and EAI 7 groups. One-way ANOVA analysis with Bonferroni test and Kruskal-Wallis test for comparisons of respiratory rates, heart rates, weights, and local cerebral blood, two-way ANOVA analysis with Bonferroni test for comparisons of escape latency in MWM, and independence-samples t-test for comparisons of passing times in MWM. Con 7: control group (7 days); EAI 7: epidural arterial implantation group (7 days); EAI-C7: epidural arterial implantation-contralateral measurement group (7 days); EAI-G7: epidural arterial implantation-gelatin sponge group (7 days); MWM, Morris water maze.

No significant differences were observed in respiratory and heart rates on the 7th day post-surgery or in the weights and local cerebral blood flow pre-surgery and 7 days post-surgery between the Con 7, EAI 7, EAI-C7, and EAI-G7 groups (*p* > 0.05, Bonferroni test, *n* = 6) (Fig. 3A-D, Supplementary Tables 1-3).

The mNSS and MWM tests were performed to investigate the effects of EAI surgery on neurological function. In the mNSS test, scores of all rats in the Con 7 and EAI 7 groups were zero, and no significant differences were observed in the escape latency (*p* > 0.05, Bonferroni test, *n* = 6 for the mNSS test, *n* = 12 for the MWM test) (Fig. 3E-G, Supplementary Table 4) and passing times (Con 7 vs EAI 7: 2.75±1.76 vs 2.25±1.48, *p* > 0.05, t-test, *n* = 12) (Fig. 3H, Supplementary Table 4) between the Con 7 and EAI 7 groups.

### Stability analysis of EAI in the regulation of brain ISF drainage and substance transport in brain ECS

**Figure 4.**
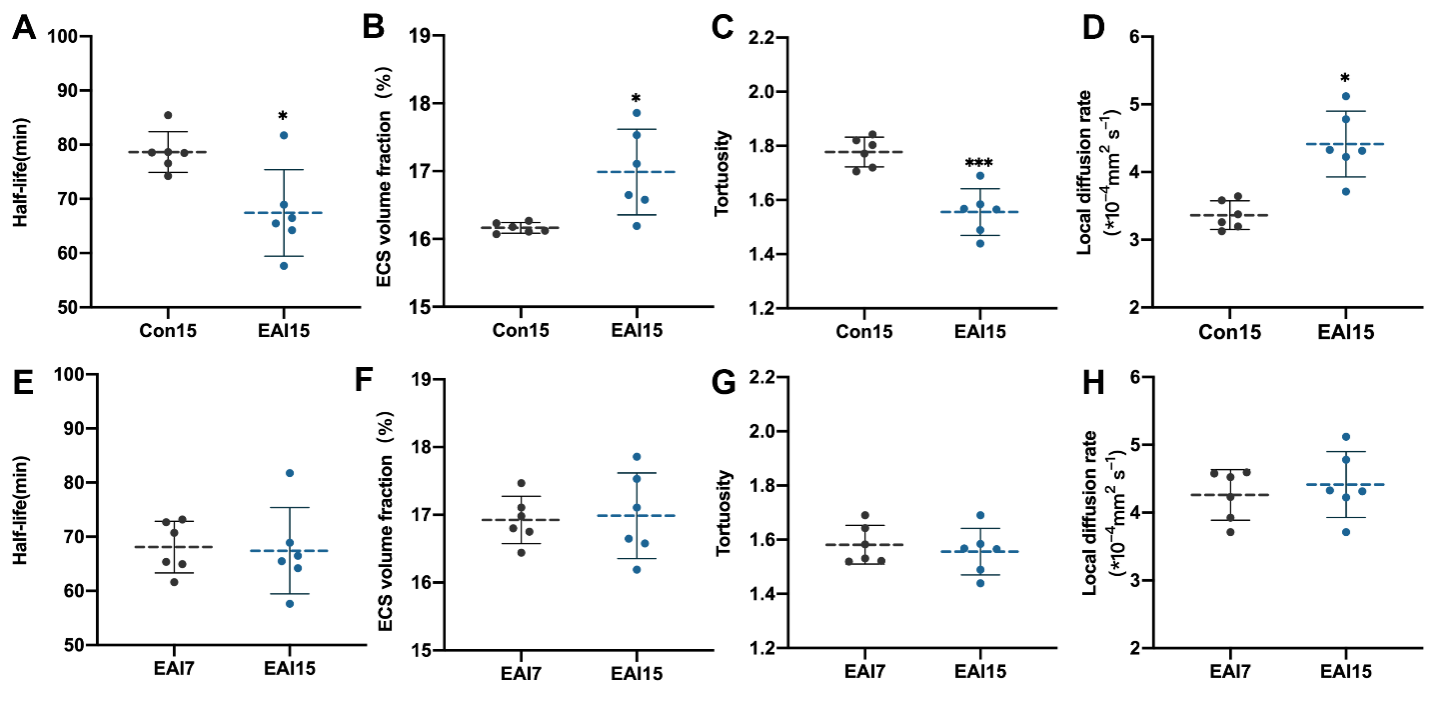
Comparison of ISF drainage and ECS structure at days 7 and 15. (A-D) Compared with the Con15 group, the EAI 15 group exhibited faster ISF drainage, higher volume fraction of brain ECS, lower ECS tortuosity, and enhanced local diffusion in Cn. (E-H) No significant differences were observed in the comparison of brain ISF drainage and ECS structural changes between the EAI 7 and EAI 15 groups in ISF drainage and ECS structure. Independent-samples t-test, *n* = 6 per group. *p < 0.05, ***p < 0.001 compared to EAI 7 group. Con 7, control group (7 days); Con 15, control group (15 days), EAI 7: epidural arterial implantation group (7 days); EAI 15, epidural arterial implantation group (15 days); ISF, interstitial fluid; ECS, extracellular space.

To further identify the stability of the surgical efficacy of EAI, we supplemented the analysis of brain ISF drainage and ECS structural parameters in rats in the EAI and control groups 15 days post-surgery (Table 1). Compared with the Con15 group, the EAI15 group exhibited faster ISF drainage (67.42 ± 7.98 min vs 78.03 ± 8.35 min, *p* < 0.05, t-test, *n* = 6) (Fig. 4A), a higher volume fraction of brain ECS (16.99 ± 0.63% vs 16.16 ± 0.08%, *p* < 0.05, t-test, *n* = 6) (Fig. 4B), lower ECS tortuosity in the Cn region (1.56 ± 0.86 vs 1.78 ± 0.55, *p* < 0.001, t-test, *n* = 6) (Fig. 4C), and enhanced local diffusion (4.41 ± 0.49 vs 3.36 ± 0.21×10^-4^/mm^2^, *p* < 0.05, t-test, *n* = 6) (Fig. 4D). No significant differences were observed in the comparison of brain ISF drainage (68.10 ± 4.75 min vs 67.42 ± 7.975 min, *p* > 0.05, t-test, *n* = 6), brain ECS volume fraction (16.93 ± 0.35% vs 16.99 ± 0.63%, *p* > 0.05, t-test, *n* = 6), tortuosity (1.58 ± 0.70 vs 1.56 ± 0.86, *p* > 0.05, t-test, *n* = 6), and local diffusion rate within the ECS (4.26 ± 0.37 vs. 4.41 ± 0.49×10^-4^/mm^2^, *p* > 0.05, t-test, *n* = 6) between the EAI 7 and EAI15 groups (Table 1, Fig. 4E-H). These results indicated that brain ISF drainage and ECS structure could be artificially and actively regulated by EAI. In particular, the enhancement effect of ISF was stable over a relatively long time.

## DISCUSSION

In this study, we established a minimally invasive method for the active modulation of cerebral ISF drainage. Epidural implantation of an external carotid artery branch resulted in accelerated ISF drainage from the Cn to the ipsilateral cortex. The elasticity and integrity of dura under EAI ensured an efficient pulsation effect on the CSF and resulted in a long-term and stable accelerated ISF drainage.

During the EAI procedure, a free branch of the external carotid artery was placed on the dura in the frontoparietal region, below which the ISF drained from the Cn, reaching the SAS to communicate with the CSF. After arriving at the CSF, the tracer molecules moved more rapidly owing to a local synchronized pulsatile compression from the implanted artery, which led to an increase in the local concentration gradient of tracer molecules between the ISF and CSF. The increase in local concentration gradient enhanced the concentration-driven diffusion of the tracer molecules from the brain ECS to CSF, which presented as an increased ISF drainage and diffusion rate. Meanwhile, acceleration of ISF drainage and ECS diffusion was accompanied by a widening of the ECS and a decrease in tortuosity. ECS contains extracellular matrix and numerous inorganic and organic molecules [8, 14]. Recent studies have shown that both the volume fraction and diffusion rate can be regulated by chemical changes in the brain ECS. Previous studies have shown that different anesthetics as well as neuronal activities could change the volume fraction of the brain ECS with the release of norepinephrine or glutamic acid in the brain [16, 23]. Other studies found that endogenous formaldehyde could affect ISF drainage in the brain ECS due to Aβ deposition [11, 24, 25]. Substance variation in the brain ECS under EAI and its potential influence on the brain ECS structure warrants further investigation.

A growing body of research indicates that abnormal deposition of materials in the brain ECS is closely associated with various brain diseases. AD is the most common cause of dementia [26]. In AD, Aβ peptides aggregate into amyloid oligomers and plaques and are involved in AD pathogenesis [27]. Indeed, impaired clearance of Aβ within the brain ECS is closely associated with AD [3]. Epilepsy is a disorder in which the brain experiences recurrent, abnormal, synchronous excitatory activity in a population of neurons that manifests as behavioral seizures. In epilepsy disorders, a moderate increase in the extracellular K^+^ concentration can enhance epileptiform activity [7]. The accumulation of proinflammatory cytokines in the brain ECS of ischemic stroke has long-term toxic effects on cortical and hippocampal neurons [28]. The safe, stable, and long-term effect of EAI on accelerating ISF drainage highlights the promising value of EAI in the treatment of these ECS blockage-related brain diseases.

## Supplementary Materials

The Supplementary data can be found online at: www.aginganddisease.org/EN/10.14336/AD.2022.0609.
