# Supplementary material for: Epidural Pulsation Accelerates the Drainage of Brain Interstitial Fluid
Source: Aging Dis. 2023 Feb 1;14(1):219–28. doi: 10.14336/AD.2022.0609 (PMC9937704; doi:10.14336/AD.2022.0609)
Supplement: Supplementary file 1 — The Supplementary data can be found online at: www.aginganddisease.org/EN/10.14336/AD.2022.0609. [file AD-14-1-219-s.pdf]

SUPPLEMENTARY DATA

Epidural Pulsation Accelerates the Drainage of Brain Interstitial Fluid

Xianjie Cai[1,2,3], Qingyuan He[2], Wei Wang[4], Chunlin Li[5], Hui Wang[1,3], Feng Yin[6], Tong Li[6], Dongsheng Kong[7], Yanxing Jia[8], Hongfeng Li[1], Junhao Yan[9], Xunbin Wei[1], Qiushi Ren[1,10], Yajuan Gao[2,3], Shuangfeng Yang[11], Huaiyu Tong[7*], Yun Peng[11*], Hongbin Han[1,2,3,12*]

© 2022. Cai X et al. Published online at http://www.aginganddisease.org/EN/10.14336/AD.2022.0609

SUPPLEMENTARY DATA

Supplementary Table 1. Heart and respiratory rate indexes (*n* = 6 per group).

Group	Heart rate (bpm)					Respiratory rate (bpm)				
	0 min	30 min	60 min	90 min	120 min	0 min	30 min	60 min	90 min	120 min
Con 7	320	324	321	322	323	58	59	61	62	58
	336	322	321	325	328	63	61	63	58	57
	323	325	324	312	331	64	60	63	57	58
	323	326	325	327	322	63	63	65	60	59
	332	321	321	321	320	64	63	62	58	58
	324	324	320	321	320	63	61	65	59	58
EAI 7	327	322	323	320	322	61	59	61	63	61
	322	321	320	321	324	62	63	62	59	61
	322	321	322	320	320	65	62	62	61	63
	320	323	324	325	323	61	63	64	62	61
	332	321	322	321	321	64	64	60	61	61
	323	332	321	315	322	62	62	63	62	62
EAI-C7	324	320	321	323	320	59	57	58	61	60
	325	323	323	322	322	62	62	63	61	61
	330	330	326	327	320	63	59	61	62	60
	322	323	321	323	323	62	64	64	62	61
	324	320	318	315	322	63	62	62	64	63
	321	322	327	321	314	61	61	60	63	62
EAI-G7	331	331	327	325	325	63	62	61	62	62
	329	321	324	328	328	62	64	62	62	62
	324	323	323	320	328	63	63	62	63	62
	328	315	328	322	327	63	61	63	61	60
	318	319	316	329	323	64	61	61	63	62
	323	320	321	329	326	62	62	59	61	63

Con 7, control group (7 days); EAI 7, epidural arterial implantation group (7 days); EAI-C7, epidural arterial implantation-contralateral measurement group (7 days); EAI-G7, epidural arterial implantation-gelatin sponge group (7 days)

© 2022. Cai X et al. Published online at http://www.aginganddisease.org/EN/10.14336/AD.2022.0609

SUPPLEMENTARY DATA

Supplementary Table 2. Weight indexes (*n* = 6 per group).

	Weights (g)											
Group	Pre-surgery						7 days post-surgery					
Con 7	259.4	254.7	256.3	257.1	263.3	260.4	280.4	273.3	276.1	281.7	286.5	279.2
EAI 7	267.1	256.3	255.3	260.2	262.4	259.6	291	277.3	278.6	286.3	284.4	281.6
EAI-C7	261.4	273.2	259.6	264.2	266.8	259.2	286.3	294.3	281.5	288.3	284.3	282.5
EAI-G7	263.4	260.5	258.5	256.6	264.1	254.5	283.6	281.5	279.5	278.3	284.6	277.4

Con 7, control group (7 days); EAI 7, epidural arterial implantation group (7 days); EAI-C7, epidural arterial implantation-contralateral measurement group (7 days); EAI-G7, epidural arterial implantation-gelatin sponge group (7 days).

Supplementary Table 3. Local cerebral blood flow indexes (*n* = 6 per group).

	Local cerebral blood flow (PU)											
Group	Pre-surgery						7 days post-surgery					
Con 7	210.60	190.30	176.40	220.80	161.20	187.10	193.90	212.50	170.80	207.20	178.20	208.90
EAI 7	175.50	193.50	217.80	187.70	201.60	210.00	182.50	187.60	197.40	217.10	198.50	214.40
EAI-C7	192.40	219.20	189.50	195.70	208.90	207.40	208.40	223.80	185.80	190.30	211.20	215.80
EAI-G7	184.50	177.20	201.80	190.60	205.30	188.40	201.50	180.50	196.80	191.70	200.60	193.70

Con 7, control group (7 days); EAI 7, epidural arterial implantation group (7 days); EAI-C7, epidural arterial implantation-contralateral measurement group (7 days); EAI-G7, epidural arterial implantation-gelatin sponge group (7 days)

Supplementary Table 4. Escape latency and passing times indexes (*n* = 12 per group).

	Escape latency (s)		Passing times	
Time	Con 7	EAI 7	Con 7	EAI 7
Day 1	48.60±11.28	50.43 ±8.52	-	-
Day 2	38.90±8.37	43.59±9.76	-	-
Day 3	25.93±7.28	31.66±7.36	-	-
Day 4	23.53±4.38	25.00±9.37	-	-
Day 5	18.75±4.27	21.00±7.25	-	-
Day 6	-	-	2.75±1.76	2.25±1.48

Con 7, control group (7 days); EAI 7, epidural arterial implantation group (7 days)

© 2022. Cai X et al. Published online at http://www.aginganddisease.org/EN/10.14336/AD.2022.0609
